# The *PoLACS4* Gene May Participate in Drought Stress Resistance in Tree Peony (*Paeonia ostii* ‘Feng Dan Bai’)

**DOI:** 10.3390/genes13091591

**Published:** 2022-09-05

**Authors:** Hongye Zhang, Shan Zhang, Meng Li, Juan Wang, Tian Wu

**Affiliations:** 1 College of Landscape Architecture and Horticulture Sciences, Southwest Forestry University, Kunming 650224, China; 2 Yunnan Functional Flower Resources and Industrialization Technology Engineering Research Center, Kunming 650224, China; 3 Institute of Ecological Development, Southwest Forestry University, Kunming 650224, China

**Keywords:** tree peony, cuticle wax, long-chain acyl-CoA synthetases, *PoLACS4*, enzymatic activity, expression analysis

## Abstract

The tree peony (*Paeonia ostii* ‘Feng Dan Bai’) has excellent drought tolerance. Although it has already been reported that the cuticle is an essential barrier against drought stress, the critical genes for cuticle resistance to drought remain unclear. However, the long-chain acyl-CoA synthetases (LACS) family of genes may be significant for the synthesis of cuticle wax. To test whether the LACS gene family is involved in cuticle response to drought stress in tree peony, we measure the thickness of cuticle stems and leaves alongside LACS enzyme activity. It is found that the cuticle thickens and the LACS enzyme increases with the maturation of stems and leaves, and there is a positive correlation between them. The LACS enzyme increases within 12 h under drought stress induced by polyethylene glycol (PEG). The transcriptome sequencing result (BioProject accession number PRJNA317164) is searched for, and a LACS gene with high expression is cloned. This gene has high homology and similarity with LACS4 from *Arabidopsis thaliana.* The gene is named *PoLACS4*. It is show to be highly expressed in mature leaves and peaks within 1 h under drought and salt stresses. All these results suggest that the LACS family of genes may be involved in cuticle response to drought stress and that *PoLACS4* is a crucial gene which responds rapidly to drought in the tree peony.

## 1. Introduction

The tree peony *Paeonia ostii* ‘Feng Dan Bai’ is a cultivated variety of Yangshan Paeonia, a wild species of the peony [1], It is an essential-oil plant; it plays a vital role in producing edible oils for human consumption and hence has broad prospects for promotion as an oil crop in China in the future [2]. Moreover, the tree peony has good tolerance to abiotic stresses [3], especially drought stress [4]. Recent studies have illustrated that ‘Feng Dan Bai’ has strong drought resistance among the five kinds of essential-oil peonies [5]. However, the desiccation tolerance mechanism in the tree peony cultivars has not yet been investigated.

We have previously shown that the tree peony can undergo phenotypic changes under harmful environmental conditions. Generally speaking, when plants lack water, they can actively adjust their morphology, growth, physiology, and biochemistry to adapt to drought stress [6,7]. Plants can reduce water loss through stomata by regulating cuticles to reduce transpiration [8] and delay the occurrence of cell dehydration stress, which plays an essential role in plant drought resistance. The cuticle is a sort of biopolymeric membrane formed by epidermal cells with a membrane-like hydrophobic lipid substance covering the surface of plants and is attached to the epidermal cell wall by the pectin layer [9]. The main functions of the cuticle include preventing the following: entry of pathogenic bacteria [10], consumption by herbivores, damage from ultraviolet rays, and mechanical damage [11,12], as well as protecting plants from drought, high salt conditions, and other abiotic stresses [13]. Under conditions of long-time drought stress, some plants may exhibit the phenomenon of cuticle thickening, and this has been found in studies of wheat [14] and *A. thaliana* [15].

Changes in cuticle defense and adaptation to drought are facilitated by waxes and cutin [16]. Waxes play an essential role in the cuticle and dominate some primary functions [17]. Drought stress induces the accumulation of waxes, prevents plant gas transpiration, and provides the main barrier to water loss [18,19]. Some wax transcription factors play critical roles in regulating wax deposits on the surface of plants [20]. They induce a noticeable increase in abiotic stress by regulating stress-responsive genes and participating in phytohormone signaling networks in plants. For example, in studies of *A. thaliana*, *AtW1N1* has been reported to resist drought stress, and *AtW1N1* was able to directly control *AtLACS,* which was confirmed to be involved in the regulation of cuticle permeability [21]. It has also been reported that *AtMYB96* adjusted to the genes of biosynthetic waxes and affected cuticular wax accumulation [22]. Very-long-chain fatty acids (VLCFAs) generate cuticular wax through acyl reduction and decarbonylation pathways [23]. Before C16 and C18 fatty acids are further extended to VLCFAs, they must be catalyzed into acyl-CoA enzymes by a critical enzyme, long-chain acyl-CoA synthases.

LACS is a family of enzymes in the acyl-coenzyme-A synthetase (ACS) family. The function of LACS has been demonstrated in *A. thaliana* where LACS affects fatty acid synthesis and catabolism [24]. This gene family comprises nine members, LACS1 to LACS9. The associated enzymes can activate 14 to 20 carbon fatty acids [25]. LACS1 and LACS2 are involved in keratin and cuticle wax synthesis and overlap functionally [26]. A recent study showed that LACS2 could regulate the permeability of plant cell cuticles [27]. Pulsifer et al. found that *A. thaliana* LACS1, LACS2, and LACS3 have very-long-chain acyl-CoA synthetase activities in vivo and are involved in plant cuticle biosynthesis [28,29]. LACS4 is involved in the synthesis and storage of membranes and the synthesis of surface waxes [30]. Bembenek et al. reported that LACS5 is an enzyme responsible for the activation of fatty acids through ligated high-energy CoA thioester bonds, and these fatty acyl-CoA conjugates are routed toward either anabolic or catabolic pathways [31]. LACS6 and LACS7 are involved in the fatty acid *β*-oxidation pathway and have the same functions in some aspects [31], as both catalyzed the fatty acids. LACS1, LACS2, LACS4, LACS8, and LACS9 all affect cuticular lipid metabolism, seed set, seed weight, and storage oil amounts [32]. LACS9 is located in the chloroplast envelope, which may be involved in synthesizing the cuticle [33]. LACS9 in rice has been identified as being involved in membrane trafficking through the secretory pathway to plastids in higher plant cells [34]. The LACS family of genes is involved in wax metabolism and this has been widely reported. Nevertheless, few reports have yet confirmed the critical genes involved in cuticle response to drought stress.

The endogenous phytohormones, ABA and GA_3_, are essential in a response to drought stress [35]. Generally, when plant cell membranes recognize a drought signal, they transmit the signal downstream through phytohormones and second messengers such as Ca^2+^ and ROS. MAPK, PPs, CDPKs, transitional information, and liberating ABA regulate transcription factors involved in different functions [36]. Transcription factors *AtbHLH68* play a role in drought responses either through ABA signaling or through the regulation of ABA homeostasis in *Arabidopsis* [37]. Liu et al. demonstrated that GA_3_ may regulate the response to drought stress in tender shoots of tea plants [35], while GA_3_ has been shown to alleviate the adverse effects of salt stress in soybean [38].

In this study, we verify that the LACS enzyme is involved in cuticle activities. We show that the LACS enzyme can respond to drought stress, and that the expression levels of critical genes vary in different organs and under different stresses. The results of this study reveal the drought resistance mechanism in the tree peony. It would provide a theoretical basis for further studies of the function of the LACS gene family in woody plants.

## 2. Materials and Methods

### 2.1. Plant Materials and Stress Treatments

In this study, we used a 2-year-old tree peony as material, which was grown in a five-gallon container in the experimental fields of the Southwest Forestry University, Kunming, Yunnan Province, China (102°10′~103°40′ E, 24°23′~26°33′ N) using open-air red-loam soil culture. The average rainfall in this area is 992.1 mm, the average annual temperature is 15.1 °C, the yearly sunshine time is 1904 h, and the average annual wind speed is 2.1 m/s. Before the experiment, all plant materials were irrigated thoroughly. Watering was then stopped to allow the soil to dry naturally. After 15 days, leaves visibly wilted and at this point plant materials were collected.

**Figure 1:**
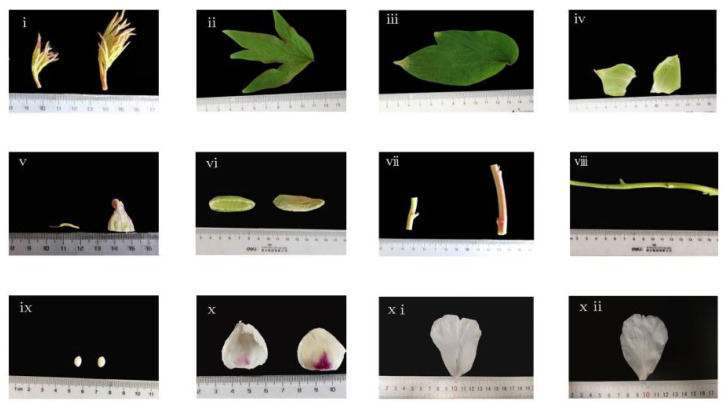
Different organs of the tree peony. (**i**) Young leaf; (**ii**) mature leaf; (**iii**) old leaf; (**iv**) calyx; (**v**) stamen and ovary; (**vi**) pod skin (30 d); (**vii**) young stem and mature stem; (**viii**) old stem; (**ix**) seeds (30 d); (**x**) young petal; (**xi**) mature petal; (**xii**) old petal.

The differentiative capacity of the tree peony has a striking distinction in each part. We picked eight plant organs from the tree peony at different developmental stages while under the drought treatment (Figure 1), including petals, leaves, stems, calyx, stamen, ovary, bean pods (30 d), and seeds (30 d). All samples were labeled and immediately frozen in liquid nitrogen and stored at −80 °C.

Samples of mature leaves were treated with 10% PEG-8000, 100 mmol/L NaCl, 100 μmol/L ABA, and 150 μmol/L GA_3_ for 0 h, 1 h, 3 h, 6 h, 12 h, 24 h, and 48 h, and three biochemical analysis replicates were performed. The samples under treatment were divided into two parts for subsequent LACS enzyme activity tests and gene expression determination. After treatment was completed, all samples were rapidly frozen in liquid nitrogen and stored at −80 °C.

### 2.2. Cuticle Thickness Measurement

The stem and leaf samples from different periods under natural drought were cut into 1 cm × 1 cm squares and immersed in FAA fixating solution (70% alcohol 90 mL, glacial acetic acid 5 ml, formaldehyde 5 mL, glycerin 5 mL), fixed for 24 h. Dehydration, transparency, wax immersion, embedding, sectioning, dewaxing and rehydration, staining, and sealing are all routine steps of paraffin sectioning. Sections were stained with 1% saffron solution for 24 h. Then, they were put into 50%, 70%, 85%, and 95% ethanol solution to wash off the red colour and stained with 1% solid green for 10 s. After concrete green staining, the slices were further dehydrated, rendered transparent and finally sealed with neutral gum. The sections were sliced using a KD-2508 rotary paraffin slicer (Zhongyi Youxin Technology Co. Ltd., Beijing, China). The sections were prepared to be observed using an Eclipse 50i light microscope (Nikon, Shanghai, China).

### 2.3. Enzyme Activity Determination

In order to verify the relationship between the LACS gene family and cuticle response to the drought stress, we measured the in vitro LACS enzyme. This experiment needs 1 g of leaf and stem samples for detection. Samples were first ground with liquid nitrogen, then 9 mL poly butylene succinate (PBS) was added and the samples were ground to just liquid, which was placed into a centrifuge for 20 min. The supernatant was taken for the ELISA test. A LACS-ELISA kit (Yanjin Biotech Co., Ltd., Shanghai, China) was used to measure the accumulation of LACS enzyme in the tree peony samples collected after different periods using a TECAN-infinite 200 pro microplate reader (Tecan (Shanghai) Trading Co., Ltd., Shanghai, China).

Leaves under treatment with 10% PEG, 100 mmol/L NaCl, 100 μmol/L ABA, and 150 μmol/L GA_3_ were taken out from the −80 °C refrigerator and the LACS enzyme changes were measured for the different periods. The LACS enzyme activity was determined according to the manufacturer’s instructions.

### 2.4. Identification and Cloning of the LACS Gene

We found a LACS gene from the transcriptome data by using real-time quantitative PCR (qRT-PCR). The expression of the LACS gene was higher than that of the other LACS genes in mature leaves under natural drought stress. The primers were designed using Blast in National Center for Biotechnology Information (NCBI), with the upstream primer LACS.F (5′-TCACTCATGGCGACATCTACC-3’) and the downstream primer LACS.R (5’-CAGATCTACTCGTAAGGAGCAA-3’). The primers were synthesized by Sangon Biotech (Shanghai, China) Co., Ltd. The PCR for the LACS gene cloning was performed using 25 µL volume containing 12 µL Taq PCR Master MixⅡ (TaKaRa, Beijing, China), 1 µL upstream primer, 1 µL downstream primer, 1 µL cDNA, and 10 µL ddH_2_O. The Labcycler 48 PCR (SensoQuest, Göttingen, Germany) protocol was 3 min at 95 °C, 35 cycles of 15 s at 95 °C, 35 cycles of 20 s at 55 °C, 35 cycles of 1 min at 72 °C, 10 min at 72 °C, and finally 10 min at 10 °C. The PCR-amplified product was cloned into the pMD18T vector (Takara, Dalian, China), transformed into *Escherichia coli* DH5α (Tiangen, Beijing, China), and also sequenced at Sangon Biotech (Shanghai) Co., Ltd. The ExPASy-Prot Param tool was used to predict and analyze the physicochemical properties of this gene. Its sequence and the LACS gene from *A. thaliana* were analyzed using the software DNAMAN.

### 2.5. RNA Extraction and qRT-PCR Analysis

Total RNA was extracted under RNase-free conditions using tree peony leaves and the Trigol reagent (Invitrogen, Carlsbad, CA, USA). cDNA fragments were synthesized using the TIANGEN Quant cDNA reverse transcription kit. The quality and integrity of RNA extracts were analyzed with 1% agar gel electrophoresis and a spectrophotometer. The qRT-PCR specific primers were designed by Primer-Blast in NCBI with the upstream primer LACS.F: (5′-CATTCGACATGGAACGCGAC-3’); and the downstream primer LACS.R: (5′-TCAGGCACTAGGCTTTATTAGCA-3), and the ubiquitin gene was used as the internal reference gene [39]. The qPCR was performed on the LightCycler 480 II (Roche, Shanghai, China). The 10 µL ChamQTMSYBR qPCR Master Mix, 0.4 µL upstream primer, 0.4 µL downstream primer, 0.4 µL ROXReferenceDye1, 1 µL cDNA, and 7.8 µL ddH_2_O were used as the qRT-PCR reaction. The qRT-PCR assays were performed according to the manufacturer’s instructions. Three replicate reactions of each sample were assayed in three dislocation curves. The CP values of target and reference genes were recorded, and the expression levels analyses of these genes were calculated by the 2^−∆∆CT^ method [40].

### 2.6. LACS Gene under PEG, NaCl, GA_3_, and ABA Treatment

To confirm whether *LACS* responds to abiotic stresses in tree peony, we treated tree peony leaves with 10% PEG, 100 mmol/L NaCl, 100 μmol/L ABA, and 150 μmol/L GA_3_ for 48 h and then measured *LACS* transcript levels using qRT-PCR.

### 2.7. Statistical Analysis

Statistical analysis was performed using Excel 2015 and IBM SPSS 26. Significant differences between groups were examined using Duncan’s multiple range test. One-way ANOVA was used for multiple comparisons. Different letters represent substantial differences at *p* < 0.05. All data are presented as mean ± standard error of the mean.

## 3. Results

### 3.1. Cuticle Thickness Is Correlated with Natural Drought Conditions

**Figure 2:**
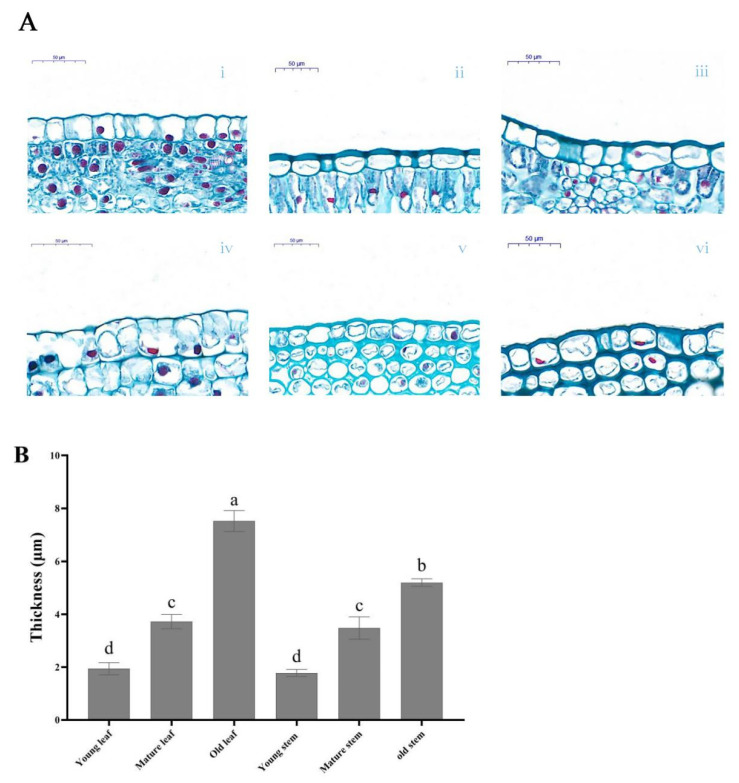
Microscopic observation of paraffin sections and thickness measurement from leaves and stems. (**A**) Paraffin section of leaves and stems. (**i**) Young leaf; (**ii**) mature leaf; (**iii**) old leaf; (**iv**) young stem; (**v**) mature stem; (**vi**) old stem. (**B**) Cuticle thickness of leaves and stems. Bars show means ± SD with three biological replicates. Different letters on the graph indicate significant differences (*p* < 0.05). The same as below.

The results of the paraffin section showed that the cuticle thickness of leaves gradually increased from young leaves to old leaves (Figure 2). The maximum thickness in the old leaves was 7.52 ± 0.39 μm, while the consistency in the young leaves was only 1.94 ± 0.23 μm. The thickness of old leaves is more than 3.8-fold that of young leaves. Cuticle thickness of stems also showed the same change from immature to old stems. The thickness of the old stems was 5.2 ± 0.14 μm, more than 2.9-fold that of the young stems 1.78 ± 0.13.

### 3.2. LACS Enzyme Activity Is Correlated with Cuticle Thickness

**Figure 3:**
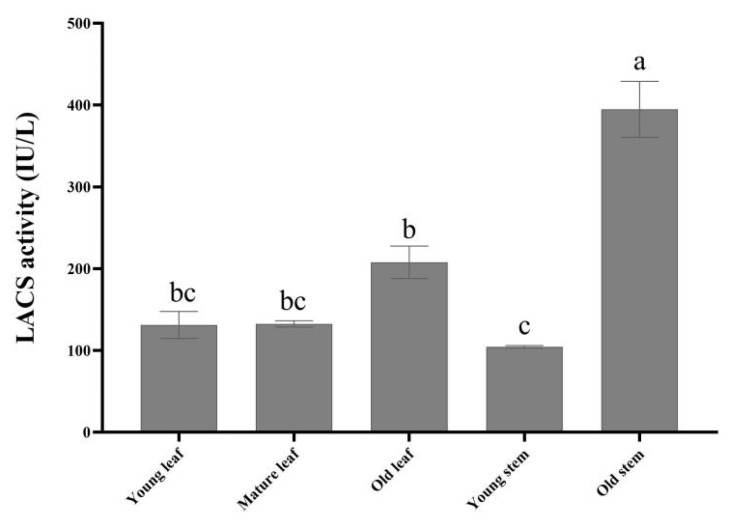
LACS enzyme activity from leaves and stems under drought.

Similar to the results for cuticle thickness, the LACS enzyme gradually increased with plant organizational maturity under drought conditions. The enzyme activity of old leaves was 1.34 times higher than that of young leaves, the enzyme activity of old stems was 3.7 times more elevated than that of young stems (Figure 3). These results were consistent with the thickness of the cuticle of stems and leaves, showing a positive correlation between the thickness of the cuticle and LACS enzyme activity in the stems and leaves from the tree peony. Therefore, it is concluded that the LACS enzyme was related to cuticle response under drought conditions.

### 3.3. LACS Enzyme Could Respond to Drought Stress

**Figure 4:**
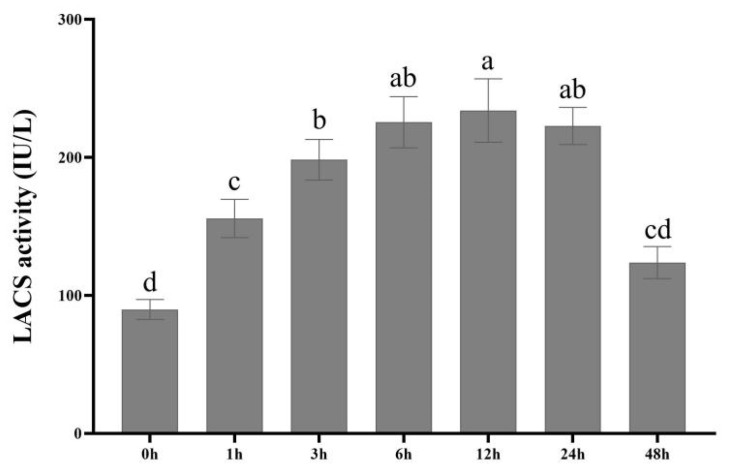
LACS enzyme activity from mature leaves induced by PEG.

The LACS enzyme activity in tree peony leaves at different times under 10% PEG treatments showed a slow upward trend from 0 h to 12 h and peaked at 12 h (Figure 4). Compared with the control, the enzyme activity under the treatment with 10% PEG increased 2–3 fold at 12 h, while it presented a downward trend at 24 h and 48 h. However, the enzyme activity at 48 h was still higher than at the control point of 0 h. These results suggest that the LACS enzyme was responsive to drought and the LACS family genes were more than likely to participate in the cuticle response to drought stress. However, except for 10% PEG, other hormones were not significant.

### 3.4. A Novel Gene from Tree Peony Is Cloned and Named PoLACS4

Based on the sequence analysis of the OFR Finder database in NCBI and subsequent protein structure analysis, PoLACS was aligned with other protein sequences with high similarity and homology in the gene database. A LACS gene with the coding region length of 1983 bp was cloned. Its starting codon was ATG, and the stop codon was TAG, encoding a protein composed of 660 amino acid residues with a molecular weight of 73.70 KD and the isoelectric point of 6.11. Meanwhile, the results of ORF were consistent with the cDNA results obtained by sequencing. The similarity of the PoLACS gene from ‘Feng Dan Bai’ with the AtLACS4 from *A. thaliana* was 76.8%.

**Figure 5:**
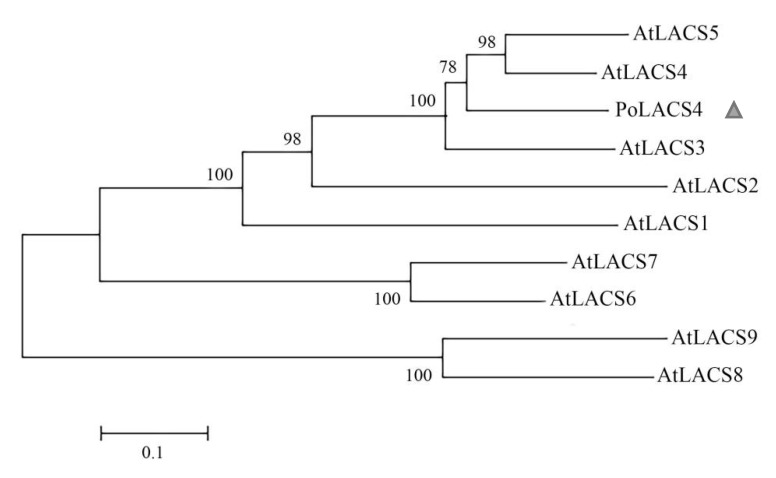
Phylogenetic analysis of PoLACS4 between ‘Feng Dan Bai’ and *Arabidopsis thaliana*.

The phylogenetic tree demonstrated that the LACS cloned from ‘Feng Dan Bai’ and the AtLACS4 from *A. thaliana* were in the same branch (Figure 5). The analysis results showed a close genetic relationship between LACS and AtLACS4, suggesting that they might have a similar function. Therefore, we named this gene *PoLACS4*. The data were submitted to the GenBank with the accession number MT745884.

### 3.5. PoLACS4 Is Highly Expressed in the Cuticle of Leaves and Petals from Tree Peony

**Figure 6:**
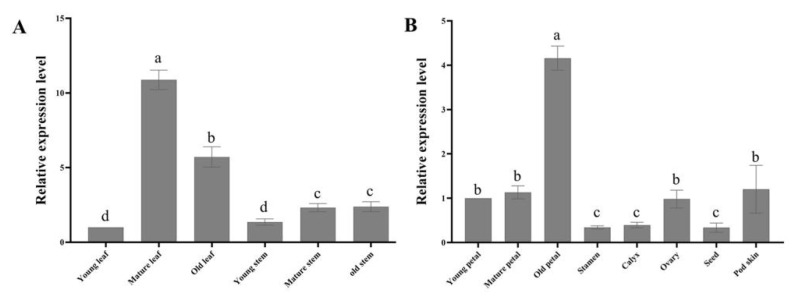
Relative expression analysis of *PoLACS4* gene from different organs. (**A**) young leaf, mature leaf, old leaf, young stem, mature stem, and old stem; (**B**) young petal, mature petal, old petal, stamen, ovary, calyx, seeds, and pod skin.

The results of the relative expression of *PoLACS4* in the different organs from the tree peony showed that the expression from the mature leaves was the highest, with 10 times that from young leaves, followed by old leaves and old petals (6-fold and 4-fold, respectively). The expression from the stamen was lower than that from other organs (Figure 6). These results indicate that *PoLACS4* positively functioned in the cuticle of tree peony leaves and petals but played little or no role in the stamen.

### 3.6. PoLACS4 Is Sensitive to Drought and Salt Stresses

**Figure 7:**
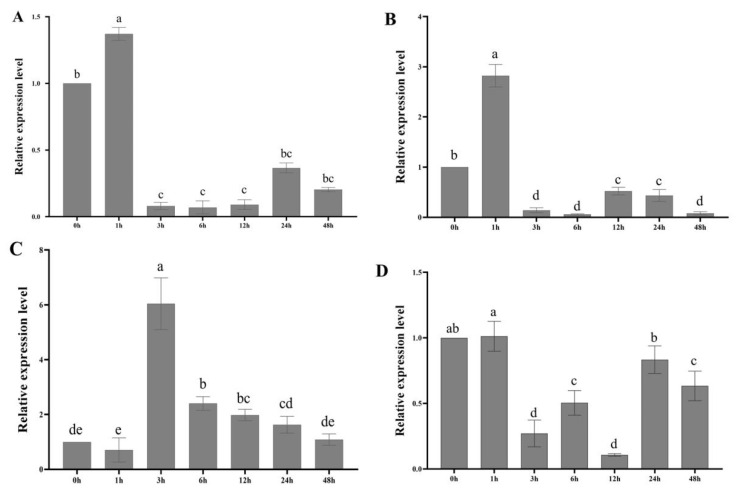
Relative expression analysis of *PoLACS4* from mature leaves under different stresses. (**A**) 10% PEG; (**B**) 150 mmol/L NaCl; (**C**) 150 μmol/L GA_3_; (**D**) 100 μmol/L ABA.

The results showed that the mature leaves of tree peony were significantly increased and reached their highest expression level at 1 h under 10% PEG treatment (Figure 7A). In comparison, the expression of *PoLACS4* at 1 h was approximately 1.3 times higher than that at the control point. The treatment with 150 mmol/L NaCl showed the same changes under the drought stress and reached the highest point within 1 h, 2.8 times higher than at 0 h (Figure 7B). These results indicate that *PoLACS4* was sensitive to drought and salt stresses. Under 150 μmol/L GA_3_ treatment, *PoLACS4* gene expression rapidly increased and peaked at 3 h, which was 6 times that at 0 h (Figure 7C). Under 100 μmol/L ABA treatment, the expression level of the *PoLACS4* gene was upregulated within 1 h and reached the maximum in the process. Subsequently, the expression level of the *PoLACS4* gene was gradually downregulated in 3 h, showing an upward trend from 6 h to 12 h and a decline slowly after 24 h (Figure 7D). *PoLACS4* was downregulated after 3 h of the 10% PEG, 150 mmol/L NaCl, and 100 mol/L ABA treatments, but the 150 μmol/L GA_3_ treatment showed upregulation after 3 h. In addition, there were no significant differences in other hormones except ABA and GA_3_.

## 4. Discussion

The tree peony is well known for its ornamental value, edible oil, and medicinal properties. However, the drought exacerbated by global climate change has limited its growing acreage [41]. Many studies have shown that the cuticle can improve plant tolerance to drought stress under water shortages. An increasing number of gene families have been discovered and studied, some essential genes have been cloned with specific pathways, and regulatory networks have been perfected. Wang et al. found that the *CmNF-YB8* gene from *Chrysanthemum morifolium* was confirmed to participate in drought resistance through cuticle accumulation and modulated stomatal movements [42]. Zhou et al. reported that the *OsWR2* gene in rice (*Oryza sativa* L.) was highly expressed in epidermal tissues and contributed to the transcriptional regulation of cuticular wax and cutin biosynthesis in rice cuticles [43]. In addition, previous studies have also found that changes in the cuticle of peony may have a regulating effect on tolerance.

The tree peony leaves were curly and had lost their green coloring under water shortage. After regular watering, the leaves quickly stretched, softened, and greened. Therefore, we speculated that the drought resistance of tree peony might be related to its cuticle. The LACS gene family is an essential gene family in fatty acid wax synthesis. However, the involvement of the LACS gene family in plant stress tolerance is rarely reported. In this study, we identified and cloned a crucial gene in the LACS family, which was identified as being a critical gene related to drought resistance, using qRT-PCR expression analysis. This identification provides a direction for further research on the tree peony stress mechanisms.

Thicker leaves are associated with greater drought tolerance, and leaf thickness can aid plants in regulating water loss [44]. Our studies found that the cuticle thickness of stems and leaves increased gradually with tissue maturity. At the same time, the LACS enzyme activity increased with tissue maturity. LACS enzyme activity was sensitive to drought and was positively correlated with tree peony cuticle thickness under drought resistance. These results indicate that LACS was involved in inhibiting drought stress under drought conditions. Therefore, it can be assumed that LACS plays a role in improving drought resistance, consistent with the results of Ahmad et al. [45]. However, further studies are needed to elucidate the detailed mechanisms underlying the interaction between the LACS gene family and the cuticle.

In this study, we compared the phylogenetic tree constructed by nine LACS from *A. thaliana* and the newly identified *PoLACS4* and concluded that the *PoLACS4* was closer to *AtLACS4*. *AtLACS4* is likely to be involved in wax synthesis and metabolism, and its function overlaps with *AtLACS1*, *AtLACS2*, *AtLACS*8, and *AtLACS9* [46]. We hypothesize that *PoLACS4* might also be involved in the synthesis and metabolism of wax in the cuticle of the tree peony.

The expression of *PoLACS4* in mature leaves was the highest in the qRT-PCR measurement of stems, leaves, and other organs of tree peony. The leaf is an important factor in drought response and is the dominant factor in wax synthesis. This is the same result as the high expression of the LACS family in apple leaf tissue [47]. These results suggest that *PoLACS4* may participate in the response of the cuticle to abiotic stress by participating in the synthesis and metabolism of wax.

We studied the expression pattern of *PoLACS4* under different stresses. Under the treatment of 10% PEG and 100 mmol/L NaCl, the expression of *PoLACS4* reached a maximum within 1 h. The result was the same as the expression of *HalACS4-5* in sunflower leaves under the treatment of 15% PEG [48]. As a critical gene, *PoLACS4* can respond rapidly to drought and salt stress in a short period and may activate a series of subsequent adaptation and resistance measures.

ABA regulates numerous gene functions in drought response by ABA-dependent and -independent pathways [49]. In a study of rice, drought and salt-stress-mediated increase in ABA levels promoted induction of *OsSWEET13* and *OsSWEET15* through ABA-responsive transcription factors *OsbZIP72* [50]. In *Arabidopsis*, it has been shown that the expression of the *AtMYB60* gene is rapidly downregulated following treatment with ABA [36]. In the study of a grapevine, *VvMYB60* showed a significant decrease in expression levels in ABA-treated samples compared to the mock-treated leaves. Conversely, *VvMYB30* did not show any change in expression after exposure to the hormone under these conditions [51].

In our study, the expression changes of *PoLACS4* were significantly downregulated in mature leaves treated with 100 μmol/L ABA. *PoLACS4* may represent a valuable target for downstream biotechnological applications. However, the expression level of *PoLACS4* reached a maximum value at 3 h after GA_3_ treatment, and the level was increased by 6 times. Liu et al. identified 211 ABA-dependent differentially expressed genes and 1118 ABA-independent DEGs under drought stress. ABA-independent DEGs were preferentially enriched in response to jasmonic acid (JA), salicylic acid (SA), and GA stimuli [49]. Our results suggest that the *PoLACS4* gene is sensitive to GA_3_, and *PoLACS4* may be regulated by exogenous GA_3_.

## 5. Conclusions

A positive relationship between the LACS enzyme and cuticle thickness exists in tree peony. High LACS enzyme activity and a thick cuticle layer are the reasons for its high drought resistance. *PoLACS4* is an essential gene for drought stress resistance in tree peony. High expression of the gene is beneficial for the study of drought tolerance in the tree peony, and can provide a theoretical basis for further research on the response mechanisms of the plant to drought stress.

## Data Availability

The data are included within the article.
